# Trajectories of Sugar-Sweetened Beverage Intake in Early Life: Evidence from a Birth Cohort Study

**DOI:** 10.3390/nu16142336

**Published:** 2024-07-19

**Authors:** Amit Arora, Kh. Shafiur Rahaman, Jinal Shashin Parmar, Adyya Gupta, Nicole Evans, Navira Chandio, Navodya Selvaratnam, Narendar Manohar

**Affiliations:** 1 School of Health Sciences, Western Sydney University, Campbelltown Campus, Locked Bag 1797, Penrith, NSW 2751, Australia; 22108248@student.westernsydney.edu.au (K.S.R.); 18467909@student.westernsydney.edu.au (J.S.P.); 12057809@student.westernsydney.edu.au (N.E.); navoselva@gmail.com (N.S.); 2 Health Equity Laboratory, Campbelltown, NSW 2560, Australia; 18154463@student.westernsydney.edu.au (N.C.); narendar.manohar@blackdog.org.au (N.M.); 3 Discipline of Child and Adolescent Health, Sydney Medical School, Faculty of Medicine and Health, The University of Sydney, Westmead, NSW 2145, Australia; 4 Oral Health Services, Sydney Local Health District and Sydney Dental Hospital, NSW Health, Surry Hills, NSW 2010, Australia; 5 Translational Health Research Institute, Western Sydney University, Campbelltown Campus, Locked Bag 1797, Penrith, NSW 2751, Australia; 6 Global Centre for Preventive Health and Nutrition, Institute for Health Transformation, School of Health and Social Development, Faculty of Health, Deakin University, Geelong, VIC 3220, Australia; adyya.gupta@deakin.edu.au; 7 Black Dog Institute, Hospital Road, Randwick, NSW 2031, Australia

**Keywords:** sugar-sweetened beverage, cohort study, preschool children, free-sugars, trajectories

## Abstract

Infancy and early childhood are periods of dietary transition. Early exposure to specific foods and the establishment of dietary habits during this period can shape long-term food preferences and have lasting effects on health. This study aimed to examine the longitudinal trajectories of sugar-sweetened beverage (SSB) intake in Australian children from birth to age 3 years and identify early-life and socioeconomic factors influencing those trajectories. Mother–infant dyads (*n* = 934) from the Healthy Smiles Healthy Kids birth cohort study were interviewed on their weekly frequency of SSB intake at 4-month, 8-month, 1-year, 2-year, and 3-year age points. Group-based trajectory modelling analysis was performed to identify trajectories for SSB intake among Australian children. A multivariable logistic regression was performed to identify the maternal and child-related predictors of resulting trajectories. The intake of SSBs showed two distinct quadratic trajectories (high and low) with age. While the two trajectories remained distinctive throughout, the SSB consumption for both groups consistently increased between 4 months and 2 years of age and subsequently stabilised. Compared to low SSB consumers (75%), the high SSB consumers (25%) were significantly more likely to be living in households with three or more children (relative risk (RR): 1.59, 95%CI: 1.02–2.48), had low maternal education (left school < year 12—RR: 1.75, 95%CI: 1.09–2.81; completed year 12—RR: 1.57, 95%CI: 1.02–2.81), and resided in highly/the most socioeconomically disadvantaged areas (highly disadvantaged—RR: 1.89, 95%CI: 1.13–3.18; most disadvantaged—RR: 2.06, 95%CI: 1.25–3.38). Children’s SSB intake patterns are established early in life as they transition from infancy to preschool age, and the trajectories of intake established during early childhood are strongly influenced by socioeconomic factors. Hence, interventions targeted to limit SSB intake and improve nutrition amongst children should occur in early life.

## 1. Introduction

Childhood obesity and dental caries are global public health issues [1,2]. In 2017–18, one in four Australian children and adolescents aged 2–17 years were overweight or obese [3]. Likewise, the National Child Oral Health Study of Australia reported that approximately 34% of children experience dental caries by the age of 5 years [4]. Both of these conditions have long-term harmful consequences [5,6] and have a risk of progressing into adolescence and adulthood [7,8]. Childhood obesity and dental caries have a multifactorial aetiology and share some common risk factors such as a diet high in free sugars, low socioeconomic status (SES), limited use of health services, and low health literacy [9,10]. Consequently, the World Health Organization (WHO) advocates for the common risk factor approach as a rational method to prevent and address all non-communicable diseases [11,12]. As a result, research in the last two decades has largely focused on tackling common risk factors such as a diet high in free sugars [13,14].

Infancy and early childhood are periods of growth, making it crucial to provide optimal nutrition and a healthy diet to ensure that the child grows and develops to their full potential [15]. It is also a phase of dietary transition, where early exposure to various foods and the establishment of dietary habits can shape long-term food preferences [16,17], particularly preferences for both healthy and less healthy dietary choices [18]. Furthermore, dietary behaviours in early life have a lasting impact on health and disease risk in later life [19]. This is particularly relevant in the context of public health concerns such as obesity and dental caries [20]. For instance, improper feeding practises during infancy may lead to overweight, which in turn is a strong predictor of obesity in childhood [7,19]. In an attempt to address this and promote a healthier lifestyle, the WHO [21] recommends limiting the consumption of free sugars to less than 10% of daily energy intake, with a further reduction to below 5%. These recommendations have been echoed globally [22,23,24,25]. Despite these efforts, many countries, including Australia, have not adopted these WHO recommendations to date within the Australian Dietary Guidelines, which currently emphasises a balanced diet without specific limits on free sugars. Moreover, the Australian and New Zealand Infant and Toddler Feeding Guidelines focus on breastfeeding, appropriate complementary feeding, and general avoidance of sugar-sweetened beverages but do not provide explicit limits on free sugar intake for infants and toddlers [26]. Furthermore, the latter has limited regulations on the composition and nutritional quality of foods, creating a challenge for parents and caregivers when making decisions for their purchase and consumption for children. This is a rising concern both nationally and internationally [27].

Sugar-sweetened beverages (SSBs) have become one of the largest sources of free sugars in the diets of children and adults [28,29]. SSBs are defined as beverages that contain various forms of sugar that contribute additional calories, which include, but are not limited to, carbonated soft drinks, energy drinks, sports drinks, electrolyte drinks, cordials, and fruit and vegetable drinks with added sugars [22,30,31]. It has been widely documented that SSBs are introduced to infants and children at an early age, especially in countries such as the United States [28] and Australia [32]. In an earlier study, we reported that 95% of infants in Sydney, Australia, were introduced to discretionary foods (energy-dense, low-nutrient) in the first year of life [33], and more specifically, 43% were introduced to SSBs before 52 weeks [32]. According to the Australian Institute of Health and Welfare (AIHW) [34], children aged 2–3 years derive approximately 8% of their daily energy intake from free sugars, with recent statistics indicating that young children still derive an excessive portion of their sugar intake from SSBs [35,36].

Studies have demonstrated the strong association between consumption of SSBs in early childhood and the risk of developing obesity and dental caries in later years [28,31]. These concerns have generated an increased interest in investigating the determinants of SSB consumption and identifying strategies to discourage and delay the introduction of SSBs among young children. A systematic review by Mazarello Paes and colleagues [37] identified key factors that exacerbate the intake of SSBs, which included the child’s personal preferences, lower SES of the family, attendance of child out-of-home care, and cultural influences that can be potentially intervened by actions through parental-, child-, and environmental-level determinants. More importantly, a study by Martin-Kerry and colleagues [38] revealed that the recommendations of the Australian Dietary Guidelines [22] to limit or avoid the consumption of SSBs have not effectively reduced their inclusion in diet during early childhood. This underscores the need for targeted efforts to reduce SSB consumption among children [39].

In recent years, several analytical approaches have been employed to examine the dietary patterns in children such as Principal Component Analysis, factor analysis, and cluster analysis [40,41,42]. These statistical techniques capture the whole diet rather than individual food or items. Despite significant focus in this area, there remains a notable gap in research that tracks longitudinal patterns or ‘trajectories’ of individual food items. Contemporary statistical approaches such as Group-Based Trajectory Modelling (GBTM) have gained attention in health and clinical sciences research over the last decade. GBTM identifies groups of individuals who follow similar patterns of health behaviours or outcomes of interest over time [43]. To the best of our knowledge, there is a dearth of evidence that has used the GBTM approach to identify longitudinal patterns of individual food items in young children [44]. Therefore, the aims of this study were the following:

1. To describe the trajectories of intake of SSBs in Australian children from birth to age 3 years;
2. To identity maternal and child-related determinants of the observed trajectories.

## 2. Methods

### 2.1. Study Population

This study used prospective data collected in the Healthy Smiles Healthy Kids (HSHK) birth cohort study in South Western Sydney [33,45,46]. In summary, the study sample comprised mothers who gave birth to babies between October 2009 and February 2010 in public hospitals located within the Sydney and South Western Sydney Local Health Districts (formerly known as the Sydney South West Area Health Service). The study involved 1035 mother–infant dyads recruited at the first post-natal visit to public hospitals in Sydney. Interpreter services and translated materials were provided for non-English speaking mothers in languages including Vietnamese, Arabic, Hindi, Assyrian, Cambodian, Cantonese, Mandarin, and Samoan. A detailed description of the study population is presented elsewhere [33,45,46].

### 2.2. Data Collection

#### 2.2.1. SSB Intake

Initially, a baseline telephone interview (at 8 weeks post-partum) was conducted to collect data on socio-demographics and infant feeding choices including breastfeeding and the use of infant formula. Subsequently, five follow-up telephone interviews were conducted when the child was 4 months, 8 months, 1 year, 2 years, and 3 years of age. At every interview, a short 32-item food frequency questionnaire (FFQ) was used to collect information on the child’s dietary habits. At each interview, mothers were asked the following question: “In the past 7 days, how often was your baby/child fed each of the following foods and/or drinks?”. A numerical response was recorded to represent the frequency of consumption of each specified food and/or drink item.

The 32 food and/or drink items listed in our questionnaire were categorised into core and discretionary foods based on the 2013 Australian Guide to Healthy Eating and the 2013 Australian Dietary Guidelines [22,47]. Core foods comprised five food groups in addition to water: grain (cereal) foods; fruit; vegetables; lean meats/poultry/fish/eggs; and dairy products. Discretionary foods were categorised into two main groups: foods high in saturated fats and foods and drinks with added sugars. Sugar-sweetened beverages, which are the focus of the current study, fall into the last category of discretionary foods. The frequency of SSB intake in the last week (continuous data) at each interview time point was used in our analysis. The questionnaire used in this study was adapted from the Iowa Fluoride Study [48], the NSW Child Health Questionnaire [49], the National Child Oral Health Survey [50], the Perth Infant Feeding Studies [51,52], and the HSHK pilot study [53].

#### 2.2.2. Potential Predictors

Several maternal and child-related factors identified from the existing literature [16,32] and considered to be potential determinants of dietary patterns in children were included in our analyses.

Maternal factors were the mother’s age at the child’s birth (in years), marital status (single or married/living with a partner), the mother’s country of birth (Australia, other English-speaking, or non-English-speaking), the mother’s level of education (<year 12, completed school, college, or university), employment status at 4 months post-partum (not working or working), the number of children in the household (1, 2, or ≥3), and area-level SES (deciles 9–10 = least disadvantaged, deciles 7–8 = less disadvantaged, deciles 5–6 = moderately disadvantaged, deciles 3–4 = highly disadvantaged, or deciles 1–2 = most disadvantaged). The SES was classified by the census-based Australian Bureau of Statistics Index of Relative Socioeconomic Advantage and Disadvantage (IRSAD) [54] using the residential postcodes of the participants.

The child’s factors were sex (male or female), duration of breastfeeding (<17 weeks, 17–25 weeks, or ≥26 weeks), and age of introduction of complementary foods (<17 weeks, 17–25 weeks, or ≥26 weeks).

### 2.3. Statistical Analysis

All statistical analyses were performed using the STATA statistical software package version 15.0 (Stata Corp LLC, College Station, TX, USA). The characteristics of the study population were extracted and summarised from the questionnaires. The mean and standard deviation (SD) were presented for continuous variables, whereas categorical variables were expressed as frequency and percentage.

#### 2.3.1. Trajectory Analysis

The longitudinal pattern of SSB intake from birth to age 3 years was examined using the Group-Based Trajectory Modelling (GBTM) method. A plug-in (PROC TRAJ) in STATA was used to construct SSB trajectories. The GBTM analyses were limited to study participants with dietary data available from at least three interview periods. A significant advantage of GBTM is its ability to handle missing data by assuming that such data are missing at random. Consequently, the model adjusts accordingly, ensuring that missing data do not affect the sample size or analytical outcome.

GBTM employs finite mixture modelling to approximate unknown trajectories among population members. It clusters individuals with similar trajectories, forming these trajectories based on maximum-likelihood estimation using a general quasi-Newton method [55]. The model selection process is guided by various parameters such as the Bayesian Information Criterion (BIC), the average posterior probability of group assignment (APPA; >0.7 for each group), Odds of Correct Classification (OCC; >5), the number of participants in each group (smallest group to be >5%), narrow confidence intervals around estimated group membership probabilities, and principal of parsimony and interpretability [56,57,58]. BIC is frequently utilised to determine the optimal number of groups that best represent the heterogeneity in the study sample’s trajectories [59]. However, BIC does not always clearly identify an optimal number of groups; hence, multiple parameters are useful. Overall, the objective of selecting the number of groups should focus on summarising the data features as parsimoniously as possible, rather than just maximising a statistical parameter.

For the present study, a Poisson-based model was selected due to the continuous distribution (count data) of the SSB frequency data at each time point. The GBTM analysis involved two steps: (1) selecting the number of groups and (2) determining the order of the polynomial defining each group’s trajectory (e.g., zero-order, linear, cubic, quadratic). A series of models with 2 to 6 groups were fitted, testing various polynomial specifications for the trajectory shapes, until the best-fitting model that had larger and more positive BIC values, acceptable APP and OCC values, tight confidence intervals around estimated group membership probabilities, a group size greater than 5%, and parsimony and interpretability was identified, hence making the final model both analytically tractable and parsimonious.

#### 2.3.2. Predictors of Trajectory Group Membership

For the second objective, multivariable logistic regression analysis was conducted to assess the association between predictors (i.e., maternal and child factors) and trajectory group membership for the ‘SSB’ group. Relative risk ratios (RRs) were generated since the dependent variable (trajectories) was dichotomous. The trajectory representing the lowest consumption group served as the reference category in the regression model. A significance level of 5% was applied to the analysis.

### 2.4. Ethics Approval

The ethics approval was granted to the former Sydney South West Area Health Service—RPAH Zone (ID number X08-0115, approval date is 19 August 2007), Liverpool Hospital, University of Sydney, and Western Sydney University. All participants signed a written consent form before participating in this study.

## 3. Results

### 3.1. Participants’ Characteristics

**Figure 1:**
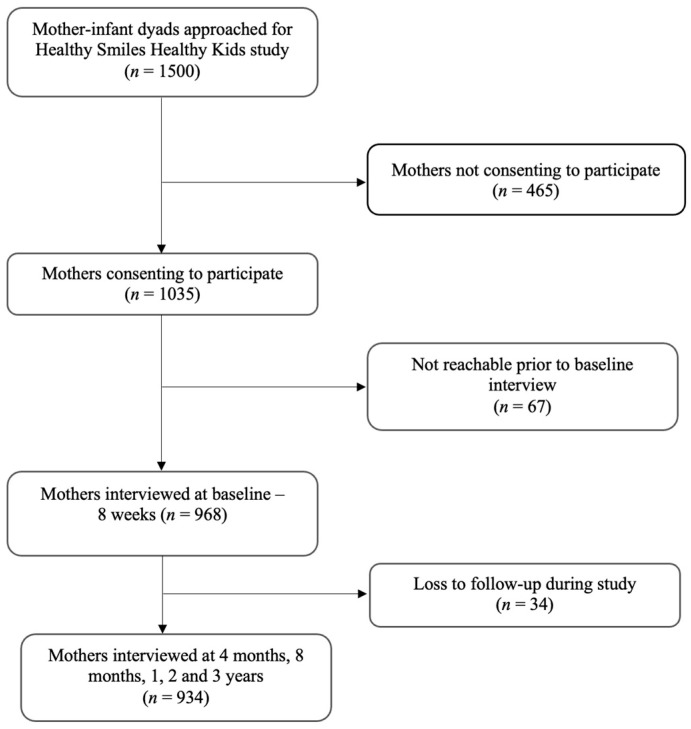
Flow chart of study sample recruitment and retention.

The flowchart in Figure 1 presents a detailed breakdown of the sample size approached, consented, withdrawals, and completed as per the eligibility criteria. Of the mother–infant dyads approached, 1035 consented to participate in the study (response rate—69%). To ensure the representativeness of the study sample, all mothers were asked three questions—age, level of education, and method of infant feeding. We then compared the socio-demographic characteristics and chosen method of infant feeding between the participating (*n* = 1035) and non-participating mothers (*n* = 465). There were no significant differences between the two groups in terms of maternal age (Chi-square (*X*^2^) = 4.75, *p* = 0.153), educational level (*X*^2^ = 6.65, *p* = 0.328), and method of infant feeding (*X*^2^ = 2.46, *p* = 0.813). Among the consenting participants, 67 mother–infant dyads either opted out or were non-contactable (seven contact attempts made) before the baseline interview. Upon excluding the lost participants to follow-up, a total of 934 participants had the SSB intake data (for at least three age points) required for this study.

Table 1 summarises the characteristics of 934 mother–child pairs in this study; 75% of children were low SSB consumers (trajectory group 1) and 25% were high SSB consumers (trajectory group 2). The average age of the mothers was 31.22 years (SD: ±5.33). In total, 60% of trajectory group 1 mothers had college/university education, while 55% in trajectory group 2 had education until year 12 or below. Nearly 90% of mothers in both groups were unemployed. Twenty-five per cent of trajectory group 2 households had more than three children, and over half of both groups lived in highly disadvantaged areas. The mean breastfeeding duration was longer in trajectory group 1 (27.48 months ±19.29) compared to trajectory group 2 (22.34 months ±19.99). Additionally, 65% of children in trajectory group 1 and 71% in trajectory group 2 were introduced to solid foods before 26 weeks.

### 3.2. Trajectories of SSB Consumption and Its Predictors

**Figure 2:**
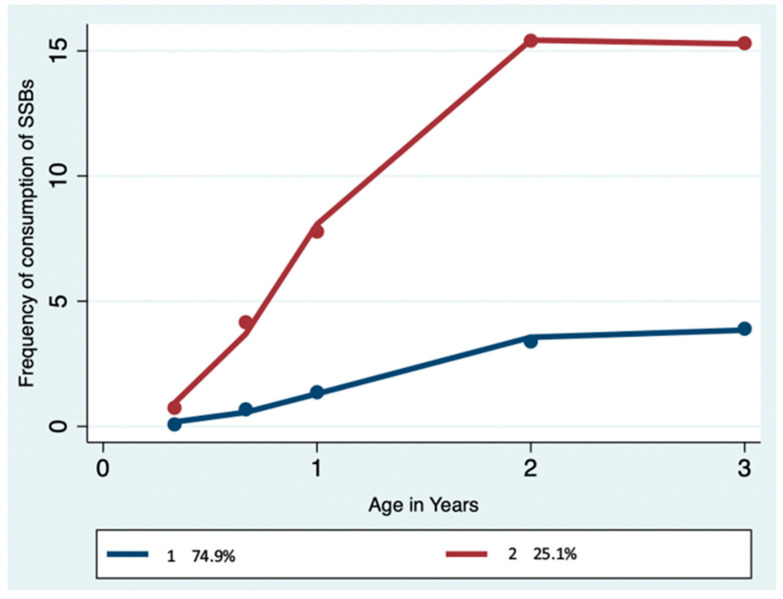
Trajectories of SSB consumption in infancy and early childhood. **Note:** SSB: Sugar-sweetened beverage; SSB trajectories: trajectory group 1—low consumers (slowly rising and late stabilising; trajectory group 2—high consumers (rapidly rising and late stabilising).

Using GBTM, two trajectories’ patterns were created for SSB intake (Figure 2). Trajectory group 1 (75%) was identified as low SSB consumers and displayed a “slowly rising and late stabilizing” consumption pattern. Trajectory group 2 (25%) was identified as high SSB consumers and displayed a “rapidly rising and late stabilizing” consumption pattern. While the two trajectories remained distinctive from 4 months to 3 years of age, the SSB consumption patterns for high and low consumers indicated an initial increase between 4 months and 2 years of age followed by gradual stability between 2 and 3 years of age (Figure 2). Table 1 presents the sample characteristics of low and high SSB consumption trajectories across maternal and child-related socio-demographic characteristics, duration of breastfeeding, and the age of introduction of solid foods.

### 3.3. Regression Analyses

Table 2 shows the adjusted regression model of the association between the SSB consumption pattern and maternal and child factors. Compared to low SSB consumers (reference trajectory group), high SSB consumers were significantly more likely to be living in households with three or more children (RR: 1.59, 95%CI: 1.02–2.48; *p* = 0.037), had low maternal education (completed year 12: RR: 1.57, 95%CI: 1.02–2.81; *p* = 0.042; left school year < 12: RR: 1.75, 95%CI: 1.09–2.81; *p* = 0.02), and resided in highly/the most socioeconomically disadvantaged areas (highly disadvantaged: RR: 1.89, 95%CI: 1.13–3.18; *p* = 0.015; most disadvantaged: RR: 2.06, 95%CI: 1.25–3.38; *p* = 0.004). Compared to low SSB consumers, no association was found between high SSB consumers and duration of breastfeeding and the age of introduction of complementary foods.

## 4. Discussion

Despite multiple short- and long-term adverse health outcomes linked to regular consumption of SSBs, children are being introduced to SSBs at an early age and excessively in many countries including Australia [28,29,30,31,60,61]. SSBs contribute to a substantial amount (approximately 4%) of energy intake (in the diet) of children aged between 2 and 3 years in Australia [35]. The present study findings show that consumption of SSBs starts at an early age (4 months). The frequency of SSB consumption continued to increase until the age of 2 years and then was stable between 2 and 3 years of age for children in trajectory group 2 (high consumers). Conversely, children in trajectory group 1 (low consumers) had a steady and slow increase in the frequency of SSB consumption between 4 months and 2 years and stabilised after 2 years of age. In a previous Australian study, the frequency of discretionary food consumption among children in Melbourne doubled between 9 and 18 months of age [17]. Unhealthy foods that are high in energy and low in nutrients make up a significant portion of “empty calories” in the diets of young children [17], and they may even replace foods that are higher in quality and/or energy value [62]. High consumption of indulgent foods like SSBs is very likely to have a negative effect on eating habits and preferences later in life [63].

Research suggests that maternal age is positively associated with healthy dietary patterns in children [64]. Some studies have reported poor-quality diets in families with younger mothers [42,65]. In contrast, our results demonstrate no association between the mother’s age and higher consumption of SSBs among their children. However, families with more than three children tend to be high SSB consumers according to our findings. In a study published in India, having a large number of family members increased the likelihood of children’s frequent consumption of SSB [66]. More importantly, previous studies highlight that younger children’s consumption of SSB was found to be prospectively correlated with that of their older siblings [67,68]. This may be true, as the eating habits of younger children are usually developed by imitation of their older siblings [69]. We did not find any association between higher consumption of SSBs and the duration of breastfeeding. However, previous studies have reported an association between breast milk intake and lower odds of consuming sweetened beverages [70,71]. The Australian Infant Feeding Guidelines and Australian Dietary Guidelines recommend avoiding the introduction of SSBs before 52 weeks and avoiding added sugars [22,23]. In our study, we did not observe any association between SSBs and the introduction of complementary foods. However, a previous study in Australia revealed that children who were given complementary foods before 17 weeks of age were nearly twice as likely to have started consuming sugar-sweetened beverages by 52 weeks compared to those who started consuming complementary foods at 17 weeks of age or later [72].

The educational status of parents determines the dietary patterns of children. Previous studies have reported that the lower educational status of parents acts as a key determinant of unhealthy eating habits [42,73]. A study conducted in the Netherlands reported that maternal education level influenced infant and young child feeding, particularly regarding SSBs [74]. This finding is in line with the findings of our study, where we found that high SSB consumers had low maternal education. The role of mothers in influencing children’s eating habits is especially important as they tend to spend more time with them and are more involved in direct feeding interactions [75]. Low-income mothers may not have a good understanding of food, which can lead to poor food literacy and consequently affect their own eating habits as well as their child’s diet [65]. For example, a qualitative study among Indigenous adults in a remote community in Northern Territory, Australia, discussed the significance of caregiver education in influencing food choices [76].

Our study suggests that high SSB consumers are more likely to reside in economically disadvantaged areas compared to low SSB consumers. Previous studies also reported an association between socioeconomic status and unhealthy dietary patterns [42,73]. A study conducted in Sydney, Australia, reported that socioeconomic status is a predictor for mothers who introduced SSBs to their infant before 52 weeks of age [32]. Socioeconomic benefits, including area-level advantages, caregiver employment, and family financial security, are linked to lower consumption of sugar-sweetened beverages (SSBs) [77]. Children from the most disadvantaged social groups often consume more discretionary foods, likely due to the affordability of these energy-rich but nutrient-poor options [78]. In contrast, families with higher socioeconomic status tend to spend more on food, which is linked to purchasing healthier options [79]. In situations of financial strain, the comparatively lower cost of sugar-sweetened beverages might make them more appealing compared to alternatives like milk, formula, fruit juices, bottled water, or milk-based drinks [77].

### 4.1. Implications for Policy

In this study, trajectories of SSB consumption were observed from as early as 4 months of age. It is therefore important to target interventions in the ante-natal and early post-partum period. Children with three or more older siblings, born to less educated mothers, and living in socioeconomically disadvantaged areas are more likely to have high SSB intake trajectories. It is pertinent to understand the factors that influence unhealthy dietary patterns such as SSB intake, as this can assist policymakers and health professionals in designing targeted interventions and providing appropriate support and practical advice to families, especially mothers as the primary caregivers of young children. Such interventions may help improve compliance with the Australian Infant Feeding Guidelines and Australian and New Zealand Infant and Toddler Feeding Guidelines providing evidence-based recommendations for promoting optimal nutrition and health for infants and toddlers, with an emphasis on breastfeeding, appropriate complementary feeding, and the avoidance of sugar-sweetened beverages [26]. Predictors of SSB intake trajectories can be used to inform the New South Wales First 2000 Days Framework, which aims to improve health and development outcomes from conception to age five by prioritising early-life nutrition and support services [80]; the New South Wales Oral Health Strategic Plan, which seeks to improve oral health through prevention, education, and access care [81]; the South Western Sydney Local Health Districts’ Growing Healthy Kids in SWS strategy, which promotes healthy eating and physical activity among children to reduce obesity and chronic diseases [82]; and the future Australian Dietary Guidelines, planned to be released in 2025.

SSBs are one of the highest contributors to free sugar intake for most Australians [34]. It is reported that a single serving of an SSB (375 mL) contains an average of 39 g of free sugars [83]. However, it is recommended to limit free sugar to 27.5 g per day to meet the WHO threshold of 5% of energy intake [84]. SSB taxes have been implemented across the world, and evaluations have demonstrated effectiveness in reducing SSB purchases and consumption [85]. However, the SSB tax has met implementation barriers in Australia [86]. Previous Australian cost-effectiveness analyses have shown that a 20% SSB tax for the prevention of obesity found a greater health benefit for disadvantaged communities and healthcare cost savings of AUD 1733M over the lifetime [87]. Likewise, an Australian study that modelled a 20% SSB tax on dental caries reported that 3.89 million decayed, missing, and filled teeth could be prevented, as well as AUD 666M in cost savings over 10 years [88]. More recently, Nguyen et al. (2023) reported lifetime societal cost savings of AUD 176.6M, healthcare cost-savings of AUD 122.5M, 1,309,211 decayed teeth averted, and 254.9 disability-adjusted life years (DALYs) averted with a three-fold health benefit for the most socioeconomically disadvantaged population [89]. Therefore, a 20% SSB tax in Australia seems to be a cost-effective strategy for health equity. Additionally, alternatives to SSBs could be promoted by targeted media advertisements as they may influence children [90].

### 4.2. Strengths and Limitations

An important strength of this study is that the same tool was consistently employed to record children’s SSB intake at regular intervals throughout a three-year span. This consistent documentation ensures an accurate capture of SSB intake over time. Moreover, the determination of age point for the first interviews was informed by scientific evidence. The frequency of SSB intake was recorded for 7 days prior to the interview at each time point, which may provide a better representation of the child’s habitual intake. This research utilised an innovative GBTM analysis, which facilitates the identification of heterogeneity in the development of SSB intake patterns at multiple time points. This method eliminates the need for subjective criteria and thresholds in defining consumer groups. Additionally, this study investigates the consumption frequency of SSBs amongst Australian children in the first three years of life, contrasting with previous Australian studies that focused only on Indigenous Australian children [77]. A substantial number of participants (*n* = 934) were retained and included in the trajectory analysis, enhancing both the precision and power of the study. Finally, incorporating a broad array of maternal and child measures may help in identifying any potential intervention strategies to target SSB consumption in the future.

The children’s SSB intake data were parent-reported, so there may be a possibility of under-reporting, social desirability bias, and/or inaccurate dietary recall. However, data collection at regular time intervals reduces the risk of recall bias and heaping of data. Although the FFQ was adapted from the well-established literature, its validity and appropriateness for culturally and linguistically diverse children needs to be considered. Nonetheless, FFQs are easy to administer and commonly used in longitudinal studies [91]. Additionally, only the frequency of SSB intake was recorded rather than both frequency and serving size/amount, so we could not capture the actual intake. There is an inherent assumption that high frequency equates to a greater amount. Our study also assumes that all mothers correctly identified all the SSBs taken by the child. Given that children’s diet is complex, it is possible that some SSBs were inadvertently missed in our questionnaire. Due to certain specific characteristics of Australian birth cohort studies and the uniqueness of certain populations, the present study findings may not be generalised to every other population. Finally, the PROC TRAJ plug-in used in GBTM analysis does not take into account the growth factor variances within each group, which theoretically may affect trajectory group membership in some cases. Nonetheless, GBTM is a valid and effective analytical tool to investigate the patterns (or trajectories) of dietary intake across the life course.

## 5. Conclusions

SSB intake in early life showed two distinct quadratic trajectories of high and low with age. While the two trajectories remained distinctive throughout, the SSB consumption for both groups consistently increased between 4 months and 2 years of age and subsequently stabilised. Unlike in the trajectory of low SSB intake, consumers representing the trajectory of high SSB intake were notably associated with residing in households with three or more children, having lower maternal education, and living in socioeconomically disadvantaged areas. Conversely, no significant relationship was found between high SSB consumption and the duration of breastfeeding or the timing of introducing complementary foods. These findings call for an urgent need to establish a dialogue between the government and other stakeholders to direct coordinated efforts towards reducing SSB consumption among children to prevent unwarranted health risks like childhood obesity and dental caries and increase long-term health and dietary benefits.

## Figures and Tables

**Table 1:** Characteristics of the study sample.

Variables	SSB Trajectories ^a,b^		
	Total Sample (*n* = 934)	Group 1 (*n* = 710)	Group 2 (*n* = 224)
**Maternal factors**			
**Maternal age** (*n* = 933) Mean ± SD	933 (31.22 ± 5.33)	31.45 ± 5.16	30.52 ± 5.79
**Maternal marital status** (*n* = 934)			
Married/Living with partner	844 (90.36%)	654 (92.11%)	190 (84.82%)
Single	90 (9.64%)	56 (7.89%)	35 (15.18%)
**Maternal education** (*n* = 934)			
University	404 (43.25%)	334 (47.04%)	70 (31.25%)
College/TAFE	170 (18.20%)	139 (19.58)	31 (13.84%)
Completed 12	192 (20.56%)	130 (18.31%)	62 (27.68%)
Left school < 12	168 (18%)	107 (15.07%)	61 (27.23%)
**Employment status** (*n* = 932)			
Not working	826 (88.63%)	623 (87.99%)	203 (90.62%)
Working	106 (11.37%)	85 (12.01%)	21 (9.38%)
**Number of children** (*n* = 934)			
1	465 (49.79%)	372 (52.39%)	93 (41.52%)
2	283 (30.30%)	208 (29.30%)	75 (33.48%)
≥3	186 (19.91%)	130 (18.31%)	56 (25%)
**Index of relative socioeconomic advantage and disadvantage** (*n* = 934)			
Decile 9–10	221 (23.66%)	192 (27.04%)	29 (12.95%)
Decile 7–8	160 (17.13%)	127 (17.89%)	33 (14.73%)
Decile 5–6	30 (3.21%)	22 (3.10%)	8 (3.57%)
Decile 3–4	220 (23.55%)	161 (22.68%)	59 (26.34%)
Decile 1–2	303 (32.44%)	208 (29.30%)	95 (42.41%)
**Child factors**			
**Child gender** (*n* = 934)			
Female	457 (48.93%)	352 (49.58%)	105 (46.88%)
Male	477 (51.07%)	358 (50.42%)	119 (53.12%)
**Duration of breastfeeding** (*n* = 932) Mean ± SD	932 (26.25 ± 19.58)	27.48 ± 19.29	22.34 ± 19.99
**Age of introduction of solid foods** (*n* = 913)			
<26 weeks	608 (66.59%)	452 (65.32%)	156 (70.59%)
≥26 weeks	305 (33.41%)	240 (34.68%)	65 (29.41%)

**Note:** ^a^ **SSB trajectories:** trajectory group 1—low consumers (slowly rising and late stabilising; trajectory group 2—high consumers (rapidly rising and late stabilising) ^b^ The total of the categories might not always add up to 934 due to missing or incomplete data for some items. ***n*:** sample size. **SSB:** Sugar-sweetened beverage. **Index of relative socioeconomic advantage and disadvantage:** deciles 9–10 = least disadvantaged; deciles 7–8 = less disadvantaged; deciles 5–6 = moderately disadvantaged; deciles 3–4 = highly disadvantaged; deciles 1–2 = most disadvantaged.

**Table 2:** Factors associated with SSB pattern trajectories in infancy and early childhood.

SSB Trajectories	Adjusted RR	95%CI		*p*
Group 1 *	(reference group)			
Group 2 **				
**Child sex**				
Female	1.00			
Male	1.11	0.81	1.52	0.520
**Maternal age (in years)**	0.98	0.95	1.01	0.208
**Maternal marital status**				
Married/Living with partner	1.00			
Single	1.31	0.78	2.17	0.303
**Number of children**				
1	1.00			
2	1.27	0.88	1.85	0.204
≥**3**	1.59	1.03	2.48	0.037
**Maternal education**				
University	1.00			
College/TAFE	0.92	0.57	1.49	0.732
Completed 12	1.57	1.02	2.44	0.042
Left school < 12	1.75	1.09	2.82	0.020
**Maternal work status**				
Not working	1.00			
Working	0.91	0.54	1.54	0.730
**Index of relative socioeconomic advantage and disadvantage**				
Decile 9–10	1.00			
Decile 7–8	1.56	0.89	2.74	0.121
Decile 5–6	1.86	0.74	4.72	0.189
Decile 3–4	1.89	1.13	3.18	0.015
Decile 1–2	2.06	1.26	3.38	0.004
**Breastfeeding duration (in months)**	0.99	0.98	1.00	0.201
**Age of introduction of solid foods**				
<26 weeks				
≥26 weeks	0.87	0.61	1.24	0.450

**Note:** * Trajectory group 1—low consumers (slowly rising and late stabilising). ** Trajectory group 2—high consumers (rapidly rising and late stabilising). **RR:** Relative risk ratio, **95%CI:** 95% confidence interval, ***p***: *p*-value (significance set at <0.05). **Index of relative socioeconomic advantage and disadvantage:** deciles 9–10 = least disadvantaged; deciles 7–8 = less disadvantaged; deciles 5–6 = moderately disadvantaged; deciles 3–4 = highly disadvantaged; deciles 1–2 = most disadvantaged.

## Data Availability

The data of this study cannot be shared publicly due to the presence of sensitive (confidential) participants’ information.
